# Post cementation sensitivity evaluation of glass Ionomer, zinc phosphate and resin modified glass Ionomer luting cements under class II inlays: An *in vivo* comparative study

**DOI:** 10.4103/0972-0707.62638

**Published:** 2010

**Authors:** V Chandrasekhar

**Affiliations:** Mamata Dental College, Khammam-507 002, AP, India

**Keywords:** Cementation, glass ionomer cement, inlay, sensitivity, zinc phosphate cement

## Abstract

**Objective:**

This study aims to compare the patient-perceived post-cementation sensitivity of class II metal restorations preoperatively, immediately after cementation, one week after cementation and one month after cementation with (1) Glass Ionomer luting cement (2) Zinc Phosphate cement and (3) Resin-modified Glass Ionomer luting cement.

**Materials and Methods:**

A total of 60 patients, irrespective of sex, in the age group of 15-50 years were selected and the teeth were randomly divided into three groups of 20 each. Twenty inlay cast restorations were cemented with three different luting cements. The criteria adapted to measure tooth sensitivity in the present study were objective examination for sensitivity. (1) Cold water test (2) Compressed air test and (3) Biting pressure test.

**Results:**

The patients with restorations cemented with Resin-modified Glass ionomer demonstrated the least postoperative sensitivity when compared with Glass Ionomer and zinc phosphate cement at all different intervals of time evaluated by different tests.

**Conclusion:**

The patients with restorations cemented with resin-modified Glass ionomer demonstrated the least postoperative sensitivity.

## INTRODUCTION

The main biological problem one encounters in restorative dentistry is the favorable environment provided for microbial growth under the restorations. Bacterial activity may result in increased pulp sensitivity, pulpal inflammation, and secondary caries.

Postoperative sensitivity problem may develop when one fails to properly diagnose the condition of the pulp before and during cavity preparation. One should be concerned about the old, leaky fillings and secondary caries. Bacteria may already be present deep in the dentine, perhaps even in a local necrotic area of the pulp. There may be no symptoms because fairly good drainage of inflammatory exudate may have been established through the thousands of tubules that open into fluid gaps and caries lesions.

During the period that a temporary restoration has been done, there would be good drainage, so there would be no discomfort. However, when the inlay is permanently cemented, the tooth may become symptomatic probably because outward drainage has been blocked, resulting in an accumulation of noxious substances in the pulp.

The main objective of cementation is to bring the preparation surface of the casting, especially its circumferential constituents, as intimately close as possible to the tooth substance.

The use of different luting materials to cement inlays and crowns has increased. Hence this study was undertaken to evaluate the postoperative sensitivity of three different luting cements, namely Glass Ionomer, Resin-Modified Glass Ionomer and Zinc Phosphate at various intervals of time.

## MATERIALS AND METHODS

An in vivo evaluation of postoperative sensitivity using three different luting materials namely Glass Ionomer luting cement (SHOFU, Tokyo, Japan), Zinc Phosphate cement (Harvard, Germany) and Resin-Modified Glass Ionomer cement (Vitremer; 3M ESPE, Germany) under class II metallic restorations preoperatively, immediately after cementation, one week after cementation and one month after cementation was conducted.

A total of 60 patients of both sexes in the age group of 15-50 years were selected. Criteria for selection of teeth for the study were (1) absence of periodontitis (2) absence of high caries index (3) absence of non carious tooth defects in adjacent teeth (4) presence of initial dental caries involving the proximal surface devoid of periodontal disease.

The teeth were randomly divided into three groups of 20 each. Group-I: 20 Inlay cast restorations cemented with Glass Ionomer Luting Cement. Group-II: 20 Inlay cast restorations cemented with Zinc Phosphate Cement. Group III: 20 Inlay cast restorations cemented with Resin-Modified Glass Ionomer cement.

The teeth to be restored were isolated during the initial cavity preparation with cotton rolls. A high speed hand piece and water spray during the cavity preparation was used and the finishing was done with slow speed micromotor hand piece. The cavity preparation was done following the principles of cavity preparation.

Direct wax pattern was made with inlay wax type I. Immediate investing with gypsum bonded investment and temporization with coltosal was done. The cast restorations were fabricated and examined under a magnifying glass for defects like porosity, rounded margins, incomplete castings, voids and metallic spurs. The castings were tried on the teeth using light pressure. The castings were finished and polished by NP alloy polishing kit.

Prepared cavity was debrided, dried and debris, if any, was removed as it could interfere with seating of casting and leakage. All the surfaces of the prepared cavity were coated with cement mix and also the impression surface of the casting. With a moderate hand pressure the casting was seated and the patient was asked to bite on pressure till the cement sets and later excess flash was removed and a protective layer of varnish was applied over the restoration tooth interface.

### Criteria of measuring tooth sensitivity

The criterion adapted to measure tooth sensitivity in the present study was objective examination and subjective evaluation. To assess the sensitivity from patient, stimulus like cold water, compressed air and biting pressure[1] were selected and standardization is made by recording the patient's response. Response of the patients was rated on a sensitivity scale of 0-3. The patient was explained about the rating system before testing. The following grades were assigned.

Grade 0 - No sensitivity

Grade 1 – Mild sensitivity

Grade 2 – Moderate sensitivity

Grade 3 – Severe sensitivity

The examinations included the following:[1]

1. **Cold water test:** In this method the tooth to be tested was irrigated with 5 cubic centimeters of ice cold water for five seconds using a plastic syringe. The test was stopped immediately if the patient experienced pain.
2. **Compressed air test:** A stream of compressed air was directed on to the tooth's facial and palatal/lingual surface for 10 seconds using an air water syringe.
3. **Biting pressure test:** Sensitivity to biting was assessed by having the patient bite firmly on the end of the cotton tipped applicator.

## RESULTS

Standard statistical techniques Students ‘*t*’ test and paired ‘*t*’ test were respectively used for comparison of mean differences and comparisons between different materials.

The mean values of sensitivity obtained by using Glass Ionomer cement, Zinc Phosphate cement, and Resin-Modified Glass Ionomer cement at various levels of observation were tabulated in Tables 1–3. Immediately after cementation for both cold water test and compressed air test, there was no significant difference (*P*<0.001) between all three groups [Table 2].

**Table 1:** Comparison of post cementation sensitivity using different cement media at different time points (Mean ± SD). Cold water test

Test	Time Points	ZnPO_4_	GI	‘*t*’**	‘*P*’ value *	ZnPO_4_	Vitremer	‘*t*’	‘*P*’	GI	Vitremer	‘*t*’	‘*P*’
Cold water	1 mm	1.55±1.00	1.55±1.05	0.00	NS	1.55±1.00	1.55±1.05	0.00	NS	1.55±1.05	1.55±1.05	0.00	NS
	1 Wk	1.85±0.99	0.80±0.95	3.42	0.01	1.85±0.99	0.30±0.47	6.37	0.001	0.80±0.95	0.30±0.47	2.14	0.05
	1 Mth	1.60±0.99	0.40±0.82	4.17	0.001	1.60±0.99	0.15±0.37	6.12	0.001	0.40±0.82	0.15±0.37	1.24	NS

* *P* = 0.05, 0.01 Significant

** Unpaired ‘*t*’ test, *P* = 0.001 Highly Significant, NS = Not Significant

**Table 2:** Comparison of post cementation sensitivity using different cement media at different time points (Mean ± SD). Compressed air test

Test	Time Points	ZnPO_4_	GI	‘*t*’**	‘*P*’ value *	ZnPO_4_	Vitremer	‘*t*’	‘*P*’	GI	Vitremer	‘*t*’	‘*P*’
Compressed air	1 mm	1.10±0.85	0.95±0.82	0.57	NS	1.10±0.85	0.95±0.83	0.57	NS	0.95±0.82	0.95±0.83	0.00	NS
	1 Wk	1.30±1.033	0.40±0.60	3.39	0.01	1.30±1.03	0.05±0.22	5.34	0.001	0.40±0.60	0.05±0.22	2.46	0.02
	1 Mth	1.35±1.04	0.00±0.00	5.55	0.001	1.35±1.04	0.05±0.22	5.48	0.001	0.00±0.00	0.05±0.22	1.05	NS

* *P* = 0.05, 0.01 Significant

** Unpaired ‘*t*’ test, *P* = 0.001 Highly Significant, NS = Not Significant

**Table 3:** Comparison of post cementation sensitivity using different cement media at different time points (Mean ± SD). Biting pressure test

Test	Time Points	ZnPO_4_	GI	‘*t*’**	‘*P*’ value *	ZnPO_4_	Vitremer	‘*t*’	‘*P*’	GI	Vitremer	‘*t*’	‘*P*’
Biting pressure	1 mm	0.35±0.59	0.25±0.44	0.61	NS	0.35±0.59	0.15±0.37	1.29	NS	0.25±0.44	0.15±0.37	0.77	NS
	1 Wk	0.20±0.52	0.15±0.49	0.31	NS	0.20±0.52	0.00±0.00	1.76	NS	0.15±0.49	0.00±0.00	1.36	NS
	1 Mth	0.10±0.30	0.50±0.22	0.61	NS	0.10±0.30	0.00±0.00	1.51	NS	0.05±0.22	0.00±0.00	1.05	NS

* *P* = 0.05, 0.01 Significant

** Unpaired ‘*t*’ test, *P* = 0.001 Highly Significant, NS = Not Significant

The levels of sensitivity values obtained at different levels of observation were significant at one week i.e. there was significant decrease in sensitivity when compared to group I and group II, group II being less sensitive and group II and group III, group III being least sensitive and group II showing maximum sensitivity. At one month there was significant difference between group I and group II, group I being less sensitive (*P*<0.001) and group II and group III, group III being less sensitive (*P*<0.001) but there was no significant difference between group I and group III.

There was no significant difference (*P*<0.001) between all the three groups at different intervals of time for biting pressure test [Table 3].

## DISCUSSION

The purpose of using luting material is not only to make the casting come in intimate contact with the tooth structure, but also to prevent postoperative sensitivity as well as an effective seal between the restoration and the tooth structure. One of the major factors influencing the longevity of luting cement is microleakage. The weakest interface is between the casting and the tooth junction which leads to breakdown of luting material, later postoperative sensitivity and pulpal pathosis.

A study by Johnson GH *et al.*,[1] compared Glass Ionomer and Zinc Phosphate as cementing medias under full cast crowns, using tests such as cold water test, compressed air test and biting pressure test for postoperative sensitivity. This study also had used the same evaluating criteria.

The reason for sensitivity immediately after cementation for all the three groups could be due to the initial acidity of the luting cement, which leads to the pulpal irritation. Smith and Ruse[2] compared Zinc Phosphate, Glass Ionomer and Polycarboxylate and found a general rise of pH for all cements during the first 15 minutes. Stanley HR[3] attributed the cause for more sensitivity to low pH and rapid penetration of its low molecular weight Phosphoric Acid molecule into dentinal tubules. The results of their study were in coordination with survey conducted by Klausner LH *et al.*[4] who concluded that Zinc Phosphate cement when used as a luting agent may contribute to post operative sensitivity more often.

One of the major disadvantages of dental cements is their failure to form an adhesive bond to the tooth and the casting surface.[5] Consequently, there is little resistance to microleakage. In a laboratory study Mccomb[6] reported a significantly greater retentive strength for inlays cemented with Glass Ionomer than with Zinc Phosphate cement. Omar R[5] conducted a comparative study of the retentive capacity of dental cementing agents and concluded that Glass Ionomers were superior to Zinc Phosphate, which may be the probable reason for decrease in postoperative sensitivity.

The antibacterial property of Glass Ionomer, which includes fluoride release during setting as stated by Desehepper EJ[7,8] may also contribute to decrease in postoperative sensitivity. Mitchens JC and Gronas[9] stated that the probable reason for the increased sensitivity of the teeth cemented with Zinc Phosphate luting cement and the Glass Ionomer cement and Resin-Modified Glass Ionomer may be due to their solubility in the oral environment. An in vivo study on disintegration of luting cements was done by Phillips RW *et al.*,[10] compared Glass Ionomer, Silcophosphate, and Polycarboxylate and Zinc Phosphate for a 12 months period and concluded that Glass Ionomer and Silicophosphate cement showed lowest disintegration.

Test conducted according to ISO 9917:1991(E). An acid erosion test showed that resin-modified Glass Ionomer(3M Vitremer) showed zero solubility. Studies done by Mitchem JC and Gronas[9] and Knibbs PJ and Walls[11] concluded that Glass Ionomer had the least solubility compared to Zinc Phosphate which may be the probable cause of sensitivity. An in vivo microleakage study of luting cements done by White SN *et al.*,[12] concluded that there was significant difference between the groups, showing that Zinc Phosphate group leaked significantly more than other groups. A survey of the academy members of operative dentistry using Glass Ionomer and Zinc Phosphate cement as a luting material for cast restorations was done. This survey showed that 46% of the members who used Glass Ionomer cements in their patients reported less postoperative sensitivity in comparison to 80% of the members who used Zinc Phosphate in their patients reported increased postoperative sensitivity.[4]

Mathis RS and Ferracane[13] stated that Resin-Modified Glass Ionomer luting cement has the lowest solubility because initial contamination by water is prevented. Glass ionomer has got higher flow than Zinc Phosphate in its initial set and therefore permits easier cementation and better marginal adaptation. Immediately after cementation there was no significant difference between all the three groups using cold water test, compressed air test and biting pressure test. One week after cementation there was a significant difference when cold water test and compressed air test were compared with Group I and Group II; Group II and Group III; and Group III and Group I. Group III had the least postoperative sensitivity and Group II had the maximum sensitivity.

One month after cementation there was a significant difference when cold water test and compressed air test were compared with Group I and Group II; Group II and Group III, there was no significant difference between Group I and Group III. Group II has the maximum sensitivity. The reasons could be higher solubility, initial acidity microleakage and hydraulic pressure during cementation.

There is a possibility of experimental error in judgment of responses clinically as there is no accurate method available to determine postoperative sensitivity. The follow-up period in this study was one month and it is felt that longer duration study may be taken up to have critical evaluation.

In the present study the better performance of Group III over Group II and Group I may be attributed to the probable reasons. It is felt that more number of samples and long-term studies are needed to have more extensive and confirmative opinion.

## CONCLUSION

The patients with restorations cemented with Resin-Modified Glass Ionomer demonstrated least postoperative sensitivity when compared to Glass Ionomer and Zinc Phosphate cement at all intervals of time evaluated by different tests.
